# Puerarin protects the fatty liver from ischemia-reperfusion injury by regulating the PI3K/AKT signaling pathway

**DOI:** 10.1590/1414-431X2024e13229

**Published:** 2024-02-19

**Authors:** Faji Yang, Hengjun Gao, Zheyu Niu, Qingqiang Ni, Huaqiang Zhu, Jianlu Wang, Jun Lu

**Affiliations:** 1 Department of Hepatobiliary Surgery, Shandong Provincial Hospital affiliated to Shandong First Medical University, Jinan, Shandong, China

**Keywords:** Puerarin, Fatty liver, Ischemia-reperfusion injury, Oxidative stress, Apoptosis

## Abstract

The incidence of non-alcoholic fatty liver (NAFLD) remains high, and many NAFLD patients suffer from severe ischemia-reperfusion injury (IRI). Currently, no practical approach can be used to treat IRI. Puerarin plays a vital role in treating multiple diseases, such as NAFLD, stroke, diabetes, and high blood pressure. However, its role in the IRI of the fatty liver is still unclear. We aimed to explore whether puerarin could protect the fatty liver from IRI. C57BL/6J mice were fed with a high‐fat diet (HFD) followed by ischemia reperfusion injury. We showed that hepatic IRI was more severe in the fatty liver compared with the normal liver, and puerarin could significantly protect the fatty liver against IRI and alleviate oxidative stress. The PI3K-AKT signaling pathway was activated during IRI, while liver steatosis decreased the level of activation. Puerarin significantly protected the fatty liver from IRI by reactivating the PI3K-AKT signaling pathway. However, LY294002, a PI3K-AKT inhibitor, attenuated the protective effect of puerarin. In conclusion, puerarin could significantly protect the fatty liver against IRI by activating the PI3K-AKT signaling pathway.

## Introduction

Hepatic ischemia-reperfusion injury (IRI) is common during liver transplantation and liver surgery, which is associated with postoperative morbidity and mortality (1). The occurrence of IRI can be affected by various factors, and microcirculatory interference induced by reactive oxygen species (ROS) greatly contributes to the pathogenesis of this injury (2). Although multiple mechanisms of liver regeneration after hepatectomy play a role after IRI, the residual hepatocytes are damaged by high levels of stress injury, and the inflammation impairs the proliferation of hepatocytes. With a solid grasp of the pathogenic mechanisms underlying IRI, various methods have been developed to prevent or treat it, including antioxidants, peptides, genes, and cells. However, their clinical effectiveness is limited by poor pharmacokinetics and non-specific biodistribution following systemic administration (3). As a leading chronic liver disease, non-alcoholic fatty liver disease (NAFLD) affects about 1.7 billion individuals globally (4). Both basic studies and clinical trials have shown that the extent of hepatic steatosis and increased vulnerability are associated with IRI (5,6). Although animal experiments have found different molecular targets to reduce liver injury, such as HO-1 inducer, renalase, and small-molecule drugs, no effective regimens are available to mitigate the IRI of NAFLD (7,8).

As an isoflavone glycoside, puerarin (8-C-β-D-glucopyranosyl-7,4-hydroxy-isoflavone) is a principal active component isolated from the commonly used Chinese herbal medicine Pueraria (9). In addition, puerarin has therapeutic efficacy in treating multiple diseases, such as stroke, diabetes, and high blood pressure in animal models (10-12). In addition, puerarin can inhibit NF-κB/JNK signaling and protect against organ injury in septic mice, leading to reduced systemic inflammation (13). Recent studies have shown that puerarin may exert a protective effect on IRI of the retina, myocardium, and cerebrum by suppressing the activation of TLR4/NLRP3 inflammasome or inhibiting autophagy (14-16). Furthermore, our recent study has illustrated that puerarin is an effective and practical regimen for NAFLD by regulating PARP-1/PI3K/AKT signaling pathway, further promoting mitochondrial function (17). However, it remains largely unclear whether puerarin plays a role in hepatic IRI, let alone NAFLD.

In this study, we aimed to explore whether puerarin could protect the fatty liver from IRI.

## Material and Methods

### Animals

Male C57BL/6J mice were provided by the Animal Center of Shandong Provincial Hospital Affiliated to Shandong First Medical University (China). Animals were bred in a specific pathogen-free facility. The animal-related protocols were ratified by the Ethics Committee of Shandong Provincial Hospital (No. 2019112), and the investigation conformed to the Guide for the Care and Use of Laboratory Animals by the US National Institutes of Health (NIH Publication No. 85-23, updated 2011).

The model of hepatic steatosis was established by feeding C57BL/6 mice (male, 3-4 weeks old) a high-fat diet (HFD) (D12492; Research Diets, USA) for 14 weeks as previously described (6). Forty mice were randomly divided into 8 groups (5 mice/group): control diet (CD; Laboratory Rodent Diet 5001, Lab Diet/PMI Nutrition International, Purina Mills LLC, USA), HFD, CD-IRI, HFD-IRI, CD-Puerarin-IRI, HFD-Puerarin-IRI, CD-Puerarin-IRI-LY294002, and HFD-Puerarin-IRI-LY294002 groups.

### Mouse hepatic IRI

Mice were intraperitoneally administered with puerarin (200 mg/kg; Sigma-Aldrich, USA) and LY294002 (25 mg/kg; Sigma-Aldrich) 15 min prior to modeling according to recent studies (14,18).

A previously described approach was used to induce 70% hepatic warm ischemia of mice (19). The left hepatic artery, portal vein, and bile duct branches and median liver lobes were clamped for 60 min. Mice were sacrificed after 6 h, and liver and serum specimens were harvested. Blood specimens were immediately subjected to biochemical analyses, including alanine aminotransferase (ALT) and aspartate aminotransferase (AST), using an automatic analyzer (Fuji, Japan). The liver tissue was sliced and fixed in 4% formalin or snap-frozen in liquid nitrogen.

### Western blotting analysis

Equal amounts of proteins were subjected to sodium dodecyl sulfate-polyacrylamide gel electrophoresis (SDS-PAGE) using 12% gels. The blots were incubated with the primary antibodies against PI3K (#4249, 1:1000, Cell Signal Technology, USA), AKT (ab179463, 1:10000, Abcam, USA), p-AKT (phospho T308, ab38449, 1:500, Abcam), and GAPDH (ab181602, 1:10000, Abcam) overnight.

### Hematoxylin-eosin staining

Paraffin liver sections (5 μm) were stained with hematoxylin and eosin (H&E) for histological evaluation of IRI based on standard pathology methods.

### Redox balance evaluation

The ROS level of liver tissues was determined using dihydroethidium (DHE, Keygen Biotech, China) based on the manufacturer's instructions. Briefly, frozen liver sections (4 μm) were incubated with 20 μM DHE in the dark at 37°C for 30 min and then counterstained with DAPI. The slides were then rinsed, mounted, and examined under an immunofluorescence microscope (Leica, Germany).

The liver tissues were homogenized, followed by centrifugation at 3000 *g*, for 10 min at 4°C. Subsequently, the supernatant was collected, and then the contents of superoxide dismutase (SOD, an enzyme that catalyzes the conversion of superoxide into hydrogen peroxide and oxygen), catalase (CAT, an enzyme that decomposes hydrogen peroxide into hydrogen and water), and malondialdehyde (MDA, an end-product generated by decomposition of arachidonic acid and large polyunsaturated fatty acids) were determined using commercial kits (Jiancheng Biology Engineering Institute, China).

### TUNEL apoptosis assays

The TUNEL assay was carried out using the one-step TUNEL apoptosis assay kit - red fluorescein (Beyotime Institute of Biotechnology, China). Briefly, frozen liver sections (4 μm) were dried at room temperature, followed by fixation in 4% paraformaldehyde for 40 min. Next, the sections were incubated in immune dyeing washing liquid (0.1% Triton X-100 in PBS) for 5 min and then labeled with 50 μL TUNEL reaction mixture, followed by incubation at 37°C for 1 h. Subsequently, the sections were counterstained with DAPI in the dark, mounted, and examined under an immunofluorescence microscope.

### Statistical analysis

GraphPad Prism software version 10.0 (USA) was adopted for statistical analysis. All results are reported as means±SE. Normally distributed variables were analyzed by one-way ANOVA followed by the Tukey test. A P-value less than 0.05 was regarded as statistically significant.

## Results

### Puerarin ameliorated liver IRI of NAFL

**Figure 1:**
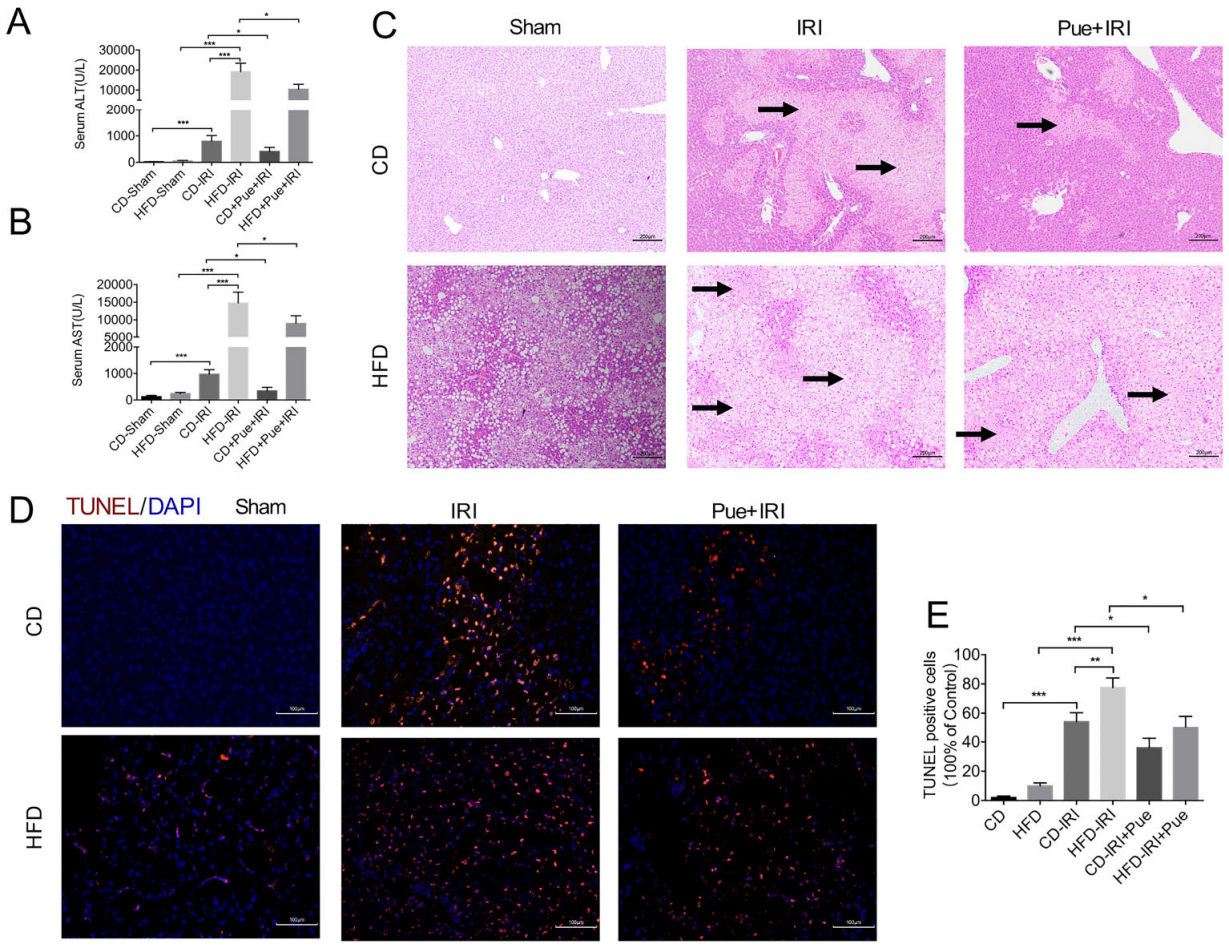
Puerarin (Pue) ameliorated liver ischemia-reperfusion injury (IRI) of non-alcoholic fatty liver (NAFLD). Serum alanine aminotransferase (ALT) (**A**) and aspartate aminotransferase (AST) (**B**) of control diet (CD)- and high-fat diet (HFD)-fed mice after 60 min of ischemia plus 6 h of reperfusion were measured with or without puerarin treatment (n=4-5 per group). **C**, Representative H&E staining of liver sections of CD- and HFD-fed mice after IR with or without puerarin treatment. Scale bars, 200 μm. Arrows indicate areas of edema, sinusoidal congestion, and necrosis. **D**, Representative immunofluorescence staining of TUNEL. Scale bars, 100 μm. **E**, The number of TUNEL positive cells was calculated (n=3 per group). Data are reported as means±SE. *P<0.05, **P<0.01, ***P<0.001. Data were analyzed by one-way ANOVA followed by the Tukey test.

To assess the impact of puerarin on hepatic IRI, we examined several biochemical indexes of liver function. Moreover, H&E staining was also conducted to evaluate liver damage. Figure 1A and B show that the levels of serum ALT and AST in HFD-fed mice were significantly higher compared with mice fed on a CD, indicating serious IRI. Puerarin pretreatment markedly decreased the serum ALT and AST levels at both doses. H&E staining showed more edema, sinusoidal congestion, and necrosis in HFD-fed mice, while the necrotic areas were reduced by puerarin pretreatment (Figure 1C). In addition, TUNEL staining showed that HFD also exacerbated hepatocyte apoptosis, a direct result of IRI damage, especially in NAFLD (Figure 1D and E). Puerarin pretreatment could also reduce apoptosis in both CD- and HFD-fed mice. Collectively, these findings suggested that puerarin played a protective role in the hepatic IRI of NAFLD.

### Puerarin reduced ROS production after IRI in NAFLD

**Figure 2:**
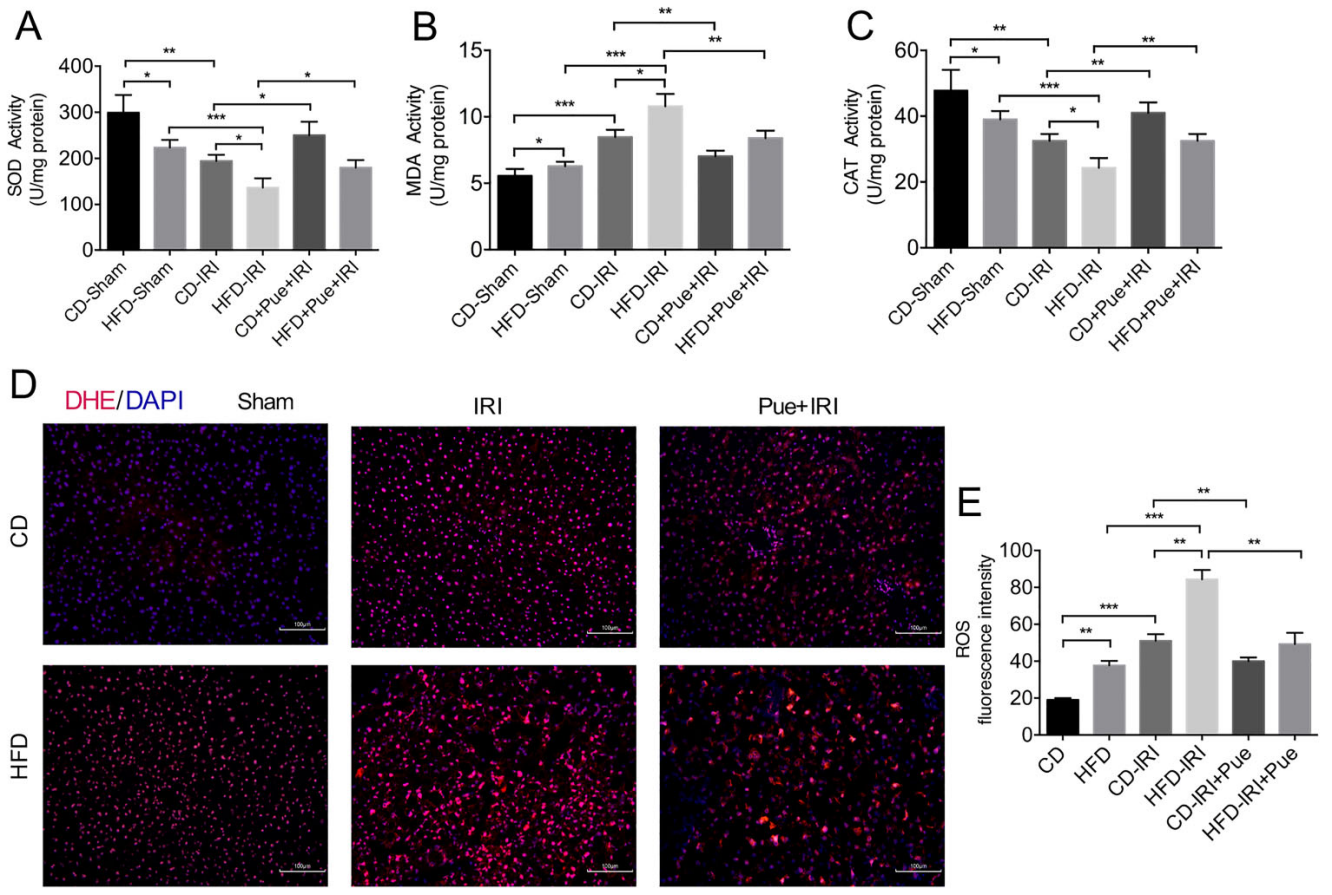
Puerarin (Pue) reduced reactive oxygen species (ROS) production after ischemia-reperfusion injury (IRI) in non-alcoholic fatty liver. **A**-**C**, Liver superoxide dismutase (SOD) activity, malondialdehyde (MDA) content, and catalase (CAT) activity (n=4-5 per group) of control diet (CD)- and high-fat diet (HFD)-fed mice after 60 min of ischemia plus 6 h of reperfusion were measured with or without puerarin treatment (n=4-5 per group). **D**, Representative images of DHE staining. Scale bars, 100 μm. **E**, Quantification of ROS fluorescence intensity (n=3 per group). Data are reported as means±SE. *P<0.05, **P<0.01, ***P<0.001. Data were analyzed by one-way ANOVA followed by the Tukey test.

Oxidative stress plays a fundamental role in regulating tissue injury during IRI. Therefore, we detected the ROS level after IRI. The MDA content was elevated in HFD-fed mice as well as the level of ROS (Figure 2B), which meant lipid peroxidation was increased. Also, Figure 2A and C shows that the activities of SOD and CAT were reduced in HFD-fed mice. After IRI, ROS production was elevated in CD-fed mice, while its elevation became more significantly pronounced in HFD-fed mice. Puerarin pretreatment significantly reduced the MDA content and increased activities of SOD and CAT in both CD- and HFD-fed mice. Moreover, DHE staining was also adopted to assess the production of ROS. Figure 2D and E shows that the DHE signal was more potent in HFD-fed mice compared with CD-fed mice after IRI. However, puerarin significantly reduced ROS production, which might be another mechanism of alleviating IRI in the fatty liver.

### Puerarin protected NAFLD from IRI through the PI3K-AKT signaling pathway

**Figure 3:**
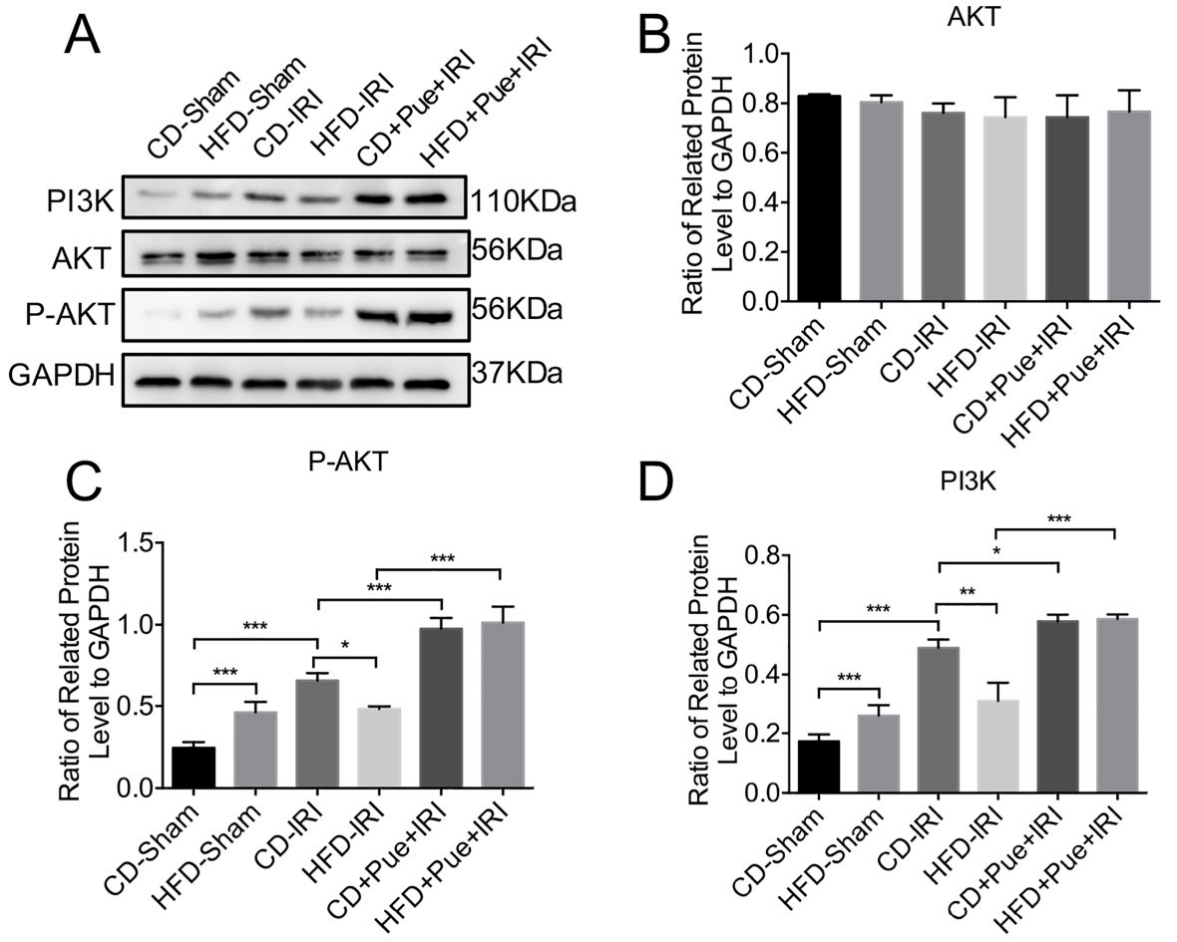
Puerarin (Pue) protected the non-alcoholic fatty liver from ischemia-reperfusion injury (IRI) through activation of PI3K-AKT signaling pathway. **A**, Immunoblot analysis of PI3K, AKT, and P-AKT of control diet (CD)- and high-fat diet (HFD)-fed mice after 60 min of ischemia plus 6 h of reperfusion with or without puerarin treatment. **B**-**D**, Protein levels were normalized to GAPDH and analyzed. Data are reported as means±SE of three independent experiments. The gels/blots and quantitative analysis were processed in parallel. *P<0.05, **P<0.01, ***P<0.001. Data were analyzed by one-way ANOVA followed by the Tukey test.

We found that puerarin exerted a protective effect on the IRI of NAFLD. However, its underlying mechanism remained largely undetermined. Our former study has found that the PI3K-AKT signaling pathway is activated in NAFLD, and puerarin plays a protective role by regulating the phosphorylation of PI3K-AKT proteins (17). Moreover, PI3K-AKT signaling also participates in hepatic IRI (20). Figure 3A-D reveals that PI3K-AKT was activated after IRI in both normal and fatty livers, while the levels of p-AKT and PI3K were lower in fatty liver than those in normal liver after IRI. Therefore, attenuated PI3K-AKT might lead to severe liver injury of NAFLD. Puerarin pretreatment could significantly increase the phosphorylation of PI3K-AKT proteins in the two above-mentioned groups. PI3K-AKT signaling pathway was activated during IRI, while the liver steatosis decreased the level of activation. Puerarin protected fatty liver from IRI through reactivating the PI3K-AKT signaling pathway.

### Suppression of PI3K-AKT attenuated the protective impact of puerarin

**Figure 4:**
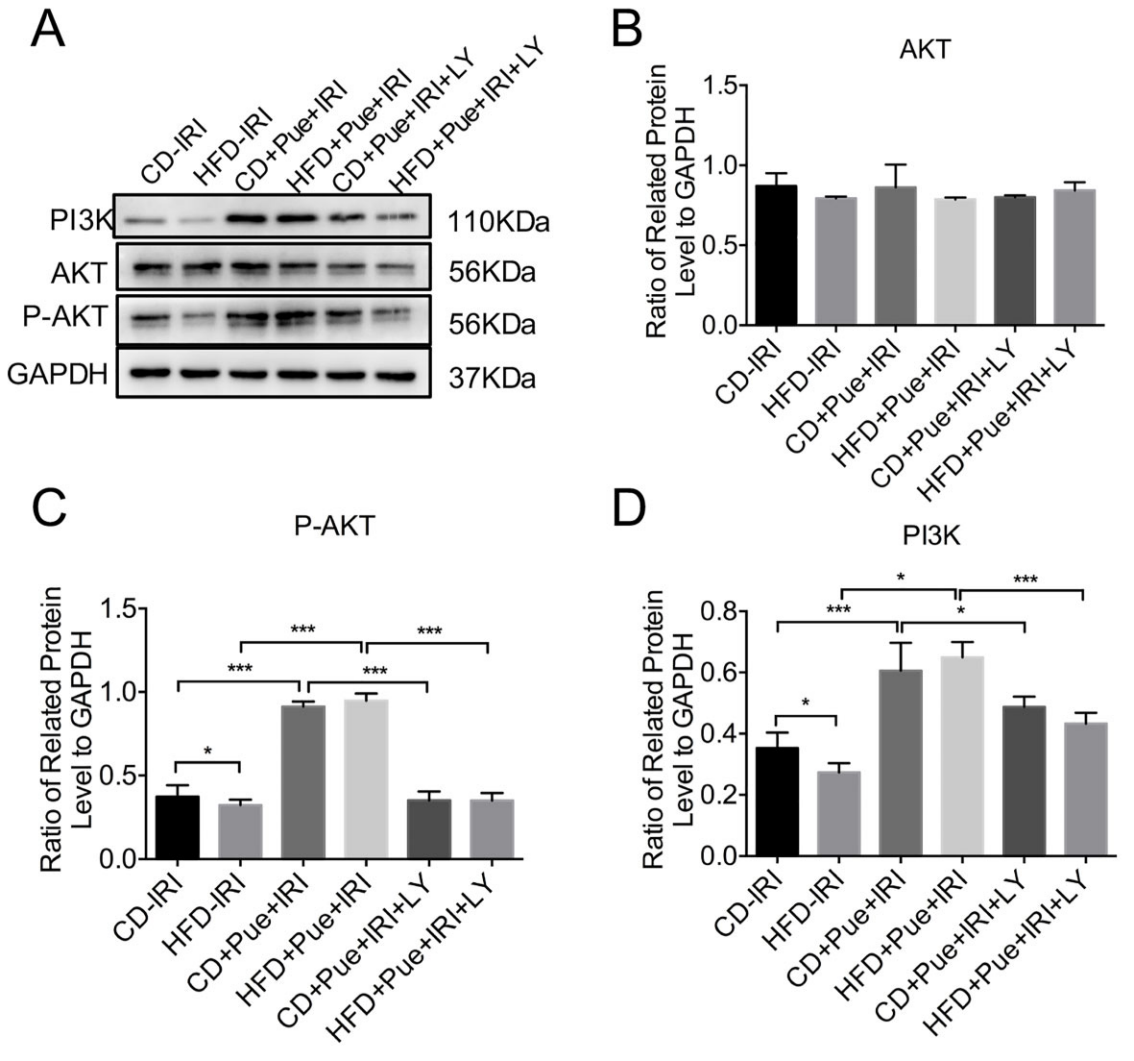
LY294002 (LY) inhibited activation of the PI3K-AKT signaling pathway induced by puerarin (Pue) treatment. **A**, Immunoblot analysis of PI3K, AKT, and P-AKT of control diet (CD)- and high-fat-diet (HFD)-fed mice after ischemia reperfusion injury (IRI) with or without puerarin or LY294002 treatment. **B**-**D**, Protein levels were normalized to GAPDH and analyzed. Data are reported as means±SE of three independent experiments. The gels/blots and quantitative were processed in parallel. *P<0.05, ***P<0.001. Data were analyzed by one-way ANOVA followed by the Tukey test.

**Figure 5:**
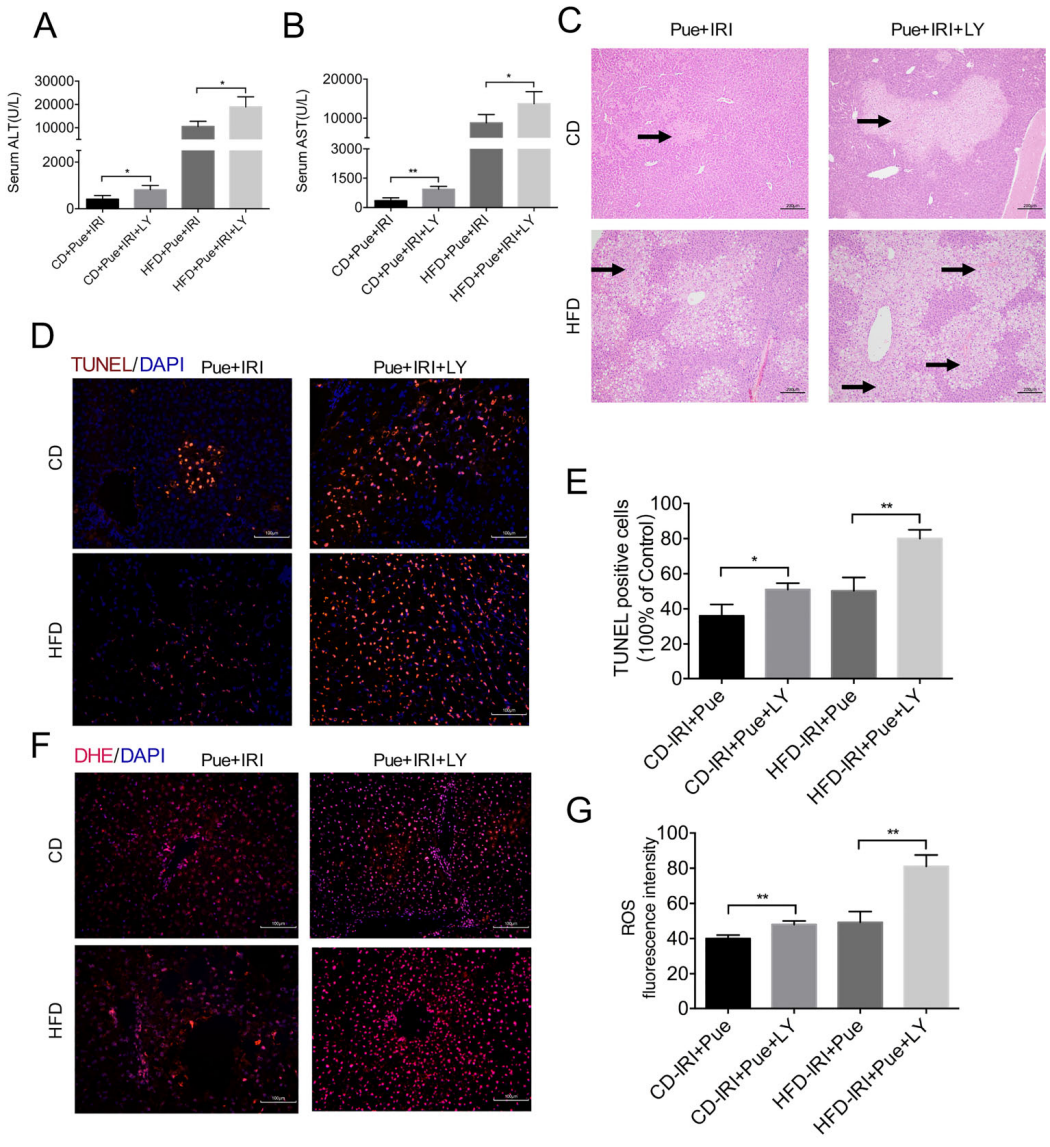
Inhibition of PI3K-AKT attenuated the protective effect of puerarin (Pue). **A** and **B**, Serum alanine aminotransferase (ALT) and aspartate aminotransferase (AST) of control diet (CD)- and high-fat diet (HFD)-fed mice after ischemia reperfusion injury (IRI) were measured with puerarin or LY294002 (LY) treatment (n=4-5 per group). **C**, Representative H&E staining of liver sections (n=3 per group). Scale bars, 200 μm. Arrows indicate areas of edema, sinusoidal congestion, and necrosis. **D**, Representative immunofluorescence staining of TUNEL. Scale bars, 100 μm. **E**, The number of TUNEL positive cells was calculated (n=3 per group). **F**, Representative images of DHE staining. Scale bars, 100 μm. **G**, Quantification of reactive oxygen species (ROS) fluorescence intensity (n=3 per group). Data are reported as means±SE. *P<0.05, **P<0.01. Data were analyzed by one-way ANOVA followed by the Tukey test.

To test the protective effect of the PI3K-AKT signaling pathway in the IRI process, we treated both CD- and HFD-fed mice with LY294002, a specific PI3K/AKT inhibitor. Elevated levels of p-AKT and PI3K induced by puerarin were significantly reduced in the LY294002 treatment group (Figure 4A-D). We also tested the liver injury. Figure 5A and B reveals that both serum ALT and AST levels were increased in the puerarin + LY294002 treatment group compared with the puerarin treatment-only group. H&E staining also showed more severe liver injury in the puerarin + LY294002 treatment group (Figure 5C). TUNEL staining indicated that LY294002 aggravated apoptosis after IRI (Figure 5D and E). Moreover, the DHE signal became more enhanced in the puerarin + LY294002 treatment group (Figure 5F and G). In conclusion, puerarin protected the IRI of the fatty liver by regulating PI3K/AKT signaling pathway, and inhibition of PI3K-AKT with LY294002 attenuated the protective effect of puerarin.

## Discussion

Hepatic IRI is a two-stage phenomenon in which blood flow to the liver is reduced, leading to cell damage and exacerbation when oxygen delivery is restored (21). Fatty liver is more sensitive to IRI, resulting in deteriorated outcomes for patients with hepatectomy and liver transplantation. NAFLD has become the most common liver disease, with a global prevalence of 25% (22). However, no available practical and straightforward approach can be used to attenuate IRI in the fatty liver. In our current investigation, IRI was more severe in NAFLD compared with the normal liver, in agreement with previous findings.

Puerarin plays a vital role in treating various diseases, including liver diseases. Recent studies have shown that puerarin can regulate alcohol and lipid metabolism, alleviating alcohol-induced hepatic steatosis in zebrafish larvae, and such a mechanism is tightly associated with the AMPKα-ACC signaling pathway (23). In addition, puerarin can deactivate the pro-inflammatory factors, up-regulate ZEB2, and inhibit NF-κB signaling pathway to reduce LPS/D-Gal-induced liver injury (24). Except for liver diseases, puerarin also plays an essential role in organ IRI. Recent studies have shown that puerarin protects against myocardial IRI by blocking inflammatory responses induced by the SIRT1/NF-κB pathway and NLRP3 inflammasome (14). Furthermore, puerarin alleviates autophagy by activating the APMK-mTOR-ULK1 signaling pathway and can be used as a therapeutic candidate for cerebral IRI (25). Thus, puerarin exerts a protective effect in both metabolic liver disease and inflammatory liver disease. In our current work, puerarin significantly reduced liver injury, as well as ROS production, in both normal and fatty livers. Our current study is the first report showing that puerarin played a protective effect on the IRI of fatty liver.

The IRI was more severe in NAFLD. The reason for this is still unclear and possibly related to the following aspects (26). First, the hepatocyte volume increases, which consequently results in the suppression of sinusoids, which supply blood to the liver. Second, in the presence of fatty liver, the beta oxidation of mitochondria is insufficient, leading to a decrease in ATP production within hepatocytes. Consequently, there is a reduction in intrahepatic energy during the process of hepatic ischemia-reperfusion, resulting in an overproduction of ROS. A recent study showed that puerarin can increase SOD in a subarachnoid hemorrhage model (27). Also, puerarin-treated ovariectomy mice exhibited higher bone density and lower levels of ROS (28). Thus, puerarin may play a role in reducing oxidative stress in this study.

As one of the most critical regulatory elements, the PI3K-AKT signaling pathway can regulate lipid metabolism (29). In the present study, PI3K-AKT was activated in NAFLD with IRI. Moreover, the PI3K-AKT signaling pathway was directly involved in the hepatic IRI process. Recent studies have found that helium preconditioning may be a promising strategy to decrease hepatic IRI by activating the PI3K-AKT pathway (30). Li et al. (31) have shown that hepatic IRI can be attenuated by inhalation of high-concentration hydrogen, and its underlying mechanism is partially associated with the A_2A_ receptor-mediated PI3K-AKT pathway activation. In addition, methyl eugenol protects the liver against IRI by activating the PI3K-AKT pathway and reducing inflammatory responses and apoptosis (32). Also, overexpression of corin protected cardiomyocytes from H_2_O_2_-induced injury by decreasing apoptosis and ROS level via activation of the PI3K/AKT and NF-κB signaling pathways and upregulating HIF-1α (33). Thus, PI3K/AKT was associated with ROS. We showed that the PI3K-AKT pathway was activated in both normal and fatty livers. Figure 3 shows that the expression of PI3K-AKT was higher in the normal liver compared with the fatty liver, leading to severe injury in fatty liver. Pretreatment with puerarin significantly activated the PI3K-AKT pathway and protected the fatty liver against IRI. Inhibition of PI3K-AKT with LY294002 attenuated the protective effect of puerarin.

In conclusion, hepatic IRI was more severe in the fatty liver than that in the normal liver, and puerarin significantly protected the fatty liver against IRI by activating the PI3K-AKT signaling pathway.
